# Clinical management of an outbreak of nutritionally variant streptococcus endophthalmitis following intravitreal bevacizumab injection

**DOI:** 10.1186/s40942-021-00287-8

**Published:** 2021-03-04

**Authors:** Alexander C. Barnes, Stephen L. Rathbun, Sanjana Kuthyar, G. Baker Hubbard, Chris Bergstrom, Steven Yeh, Mohan N. Iyer

**Affiliations:** 1 Uveitis and Vitreoretinal Surgery, Emory Eye Center, Emory University School of Medicine, Atlanta, GA USA; 2 Department of Epidemiology and Biostatistics, College of Public Health, University of Georgia, Athens, Georgia 30602 USA; 3 Retina Consultants of Carolina, 1126 Grove Road, Greenville, SC 29605 USA; 4 Uveitis and Retina Service, Truhlsen Eye Institute, University of Nebraska Medical Center, Omaha, NE USA; 5 Athens Retina Center, 2705 Jefferson Rd, Athens, GA 30607 USA

**Keywords:** Intravitreal injections, Endophthalmitis, Outbreak, Disease cluster, Nutritionally-variant streptococcus, Granulicatella

## Abstract

**Background:**

The management of an outbreak of endophthalmitis associated with intravitreal bevacizumab represents a challenging real-time process involving identification of cases, treatment and mitigation measures during the outbreak. We summarize the clinical presentation and management of a cluster of endophthalmitis cases from contaminated bevacizumab, in addition to mathematical probabilistic assessment of the number of cases that define an outbreak.

**Methods:**

A retrospective study was conducted to assess the management of an endophthalmitis outbreak after intravitreal bevacizumab (IVB) administration. Demographic data, clinical information, individual patient management and public health reporting measures were reviewed. Outcomes of patients who received prophylactic antibiotics for endophthalmitis prevention were also reviewed. Binomial tail probability calculations were performed to determine the likelihood of clusters of endophthalmitis that could inform when an outbreak was evolving that would warrant more public health notification measures and communication.

**Results:**

Forty-five eyes of 42 patients who received IVB from a single batch were reviewed. Four cases of endophthalmitis from *Granulicatella adiacens*, a nutritionally-variant Streptococcus species, were treated successfully with intravitreal antibiotics ± vitrectomy. Thirty-four of the remaining 41 eyes were treated with prophylactic intravitreal vancomycin with no additional cases of endophthalmitis. Outbreak management also included CDC, ASRS and public health authority notification. Binominal tail probabilities demonstrated the rarity of clusters from a single batch (i.e. ~ 1/10,000 for 2 cases; 1/2 million for 3 cases). However, given the U.S. scale of IVB administration, there is an 87% chance of a cluster ≧ 2 and a 1% chance of a cluster ≧ 3 cases annually, which may guide outbreak management. A process diagram was developed to incorporate patient management and public health measures when an outbreak is suspected.

**Conclusion:**

Intravitreal antibiotics and vitrectomy were effective in the individual management of cases of endophthalmitis, and no serious adverse events occurred with prophylactic intravitreal vancomycin for at-risk eyes. Best practices for outbreaks should be evaluated, given their likelihood within the U.S. and the sight-threatening consequences of endophthalmitis.

## Background

Endophthalmitis after intravitreal anti-vascular endothelial growth factor (anti-VEGF) injections is a severe, vision-threatening complication with a reported incidence ranging from 0.02 to 0.05% [1–5]. Outbreaks of bacterial endophthalmitis following anti-VEGF injections may result in vision loss from vitreous opacity, retinal detachment, and in particularly severe cases, globe loss due to infectious, inflammatory and fibrotic processes[6–9]*.*

*Streptococcus* endophthalmitis can be particularly severe with recent case series reporting severe vision loss ranging from 44 to 92% [6–8]. In one series of 12 patients who developed S. mitis/oralis endophthalmitis, 7 of 12 patients (58%) required enucleation or evisceration and showed a range of histopathologic changes that included retinal detachment, fibrous proliferation and cyclitic membrane formation [9]. Given the potential for vision- and globe-threatening sequelae of endophthalmitis in outbreak situations, anticipation of these rare events requires planning and rapid decision-making based on risk assessment and real-time data.

We report the clinical features, management, and outcomes of an endophthalmitis cluster following intravitreal bevacizumab injections. All patients from one center received intravitreal injections of bevacizumab from a single lot through a compounding pharmacy. A summary of the demographic information, clinical features of the four patients, and Centers for Disease Control Prevention (CDC) investigation of clinic practices and the compounding pharmacy was described previously [10]. In this report, we report the detailed clinical presentation, management and follow-up, as well as the decision-making. We also report statistical analyses to better understand the likelihood of clusters of endophthalmitis based on batching practices in the United States.

## Methods

Medical charts of all patients who received intravitreal bevacizumab over a three-day period from March 4th to March 6th, 2013 from a single center were retrospectively reviewed. Data collected included patient age, diagnoses, visual acuities, vitreous culture results, medical and surgical treatment, and visual acuities during follow-up.

Descriptive statistics were reported as frequencies or medians with interquartile ranges as appropriate. Binomial tail probabilities were computed based on a batch size of 70, the typical of the number of doses available from a single 100 mg vial of the drug. Statistical analyses were performed using SAS software version 9.4 (Cary, NC). The study was approved by the Institutional Review Board of the St. Mary’s Hospital, Athens, Georgia.

## Results

A total of forty-five intravitreal bevacizumab injections (1.25 mg/0.05 ml) were performed between March 4 and March 6, 2013 at a single center. Specifically, 28 patients received bevacizumab on March 4, 2013, 12 patients on March 5, 2013, and five patients on March 6, 2013. Three patients received bilateral bevacizumab injections with administration of medication for the first eye on March 4, 2013 and the fellow eye on March 6, 2013. During this three-day period, four patients presented with clinical endophthalmitis after administration of the bevacizumab injections. Patients were urgently contacted by telephone, promptly examined and treated with intravitreal antibiotics when clinical endophthalmitis was suspected. Given the rapidity of onset and severe presenting visual acuity in patients who developed clinical signs of endophthalmitis, subsequent patients who were evaluated were offered prophylactic intravitreal vancomyin injection (1 mg/0.1 ml) following the preliminary identification of a gram-positive organism by the microbiology laboratory.

### Demographic information and clinical background

The cohort of patients included 24 men (57%) and 18 women (43%) with a median age of 82 years (IQR 72–85 years). The indications for treatment with anti-VEGF injection included wet age-related macular degeneration (34 patients, 81%), diabetic macular edema (6 patients, 14%), proliferative diabetic retinopathy (1 patient, 2%), and branch retinal vein occlusion (1 patient, 2%). Of the 70 syringes in the lot prepared by the same pharmacy on February 13, 2013, 24 injections had been performed two weeks earlier with no adverse events.

### Description of cases

Four of the 28 patients who received intravitreal bevacizumab on March 4, 2013 presented with endophthalmitis. The first case presented on post injection day 2 (March 6, 2013), two cases presented on post injection day 3 (March 7, 2013), and one case presented on day 4 (March 8, 2013). The four patients who developed endophthalmitis represented 9.5% of the 42 patients treated with intravitreal bevacizumab during the three day period. Their clinical presentation, treatment and clinical course are summarized in Table 1 with details of their clinical course and management as follows.

**Table 1:** Characteristics of patients presenting with endophthalmitis

Case No./Age/Sex	Baseline vision	Vision when diagnosed with endophthalmitis	Days to presentation	Vitrectomy	Final Visual Acuity, (Follow-up Time since endophthalmitis, months)
1/74/F	20/20	HM @ 6 ft	2	N	20/25 (3)
2/85/M	20/600	LP	3	Y	20/800 (3)
3/84/M	20/40-2	20/400	3	Y	20/60-2 (3)
4/62/F	20/20-2	20/80	4	Y	20/25-2 (9)

HM Hand motions, LP Light perception

#### Case 1

A 74-year-old woman presented two days after receiving an intravitreal bevacizumab injection with decreased vision, eye pain, inflammation and fibrinous reaction in the anterior chamber without hypopyon. Fundus examination showed dense vitreous cell and haze with a poor view of the retina. Scattered retinal hemorrhages were observed. An emergent vitreous tap and injection of vancomycin (1 mg/0.1 cc) and ceftazidime (2.25 mg/0.1 cc) were performed in the clinic on March 6, 2013. Gram stain results revealed gram-positive rods with cultures pending for seven days. On March 9, 2013 (post injection day five), visual acuity declined to hand motions at 1.5 feet and a B-scan ultrasound showed increased vitreous debris. The patient underwent repeat intravitreal injection of vancomycin (1 mg/0.1 cc). There was gradual resolution of vitreous inflammation and improvement of vision over the next six weeks.

#### Case 2

An 85-year-old man presented with decreased vision and eye pain on March 7, 2013, 3 days after receiving an intravitreal bevacizumab injection. His visual acuity on presentation was light perception only. Dense vitreous inflammation precluded a view of the ocular fundus. Given the severity of acute vision loss, an emergent pars plana vitrectomy with intravitreal injection of antibiotics (vancomycin (1 mg/0.1 cc) and ceftazidime (2.25 mg/0.1 cc)) was performed. The vitreous inflammation resolved over a one-month period.

#### Case 3

**Fig. 1:**
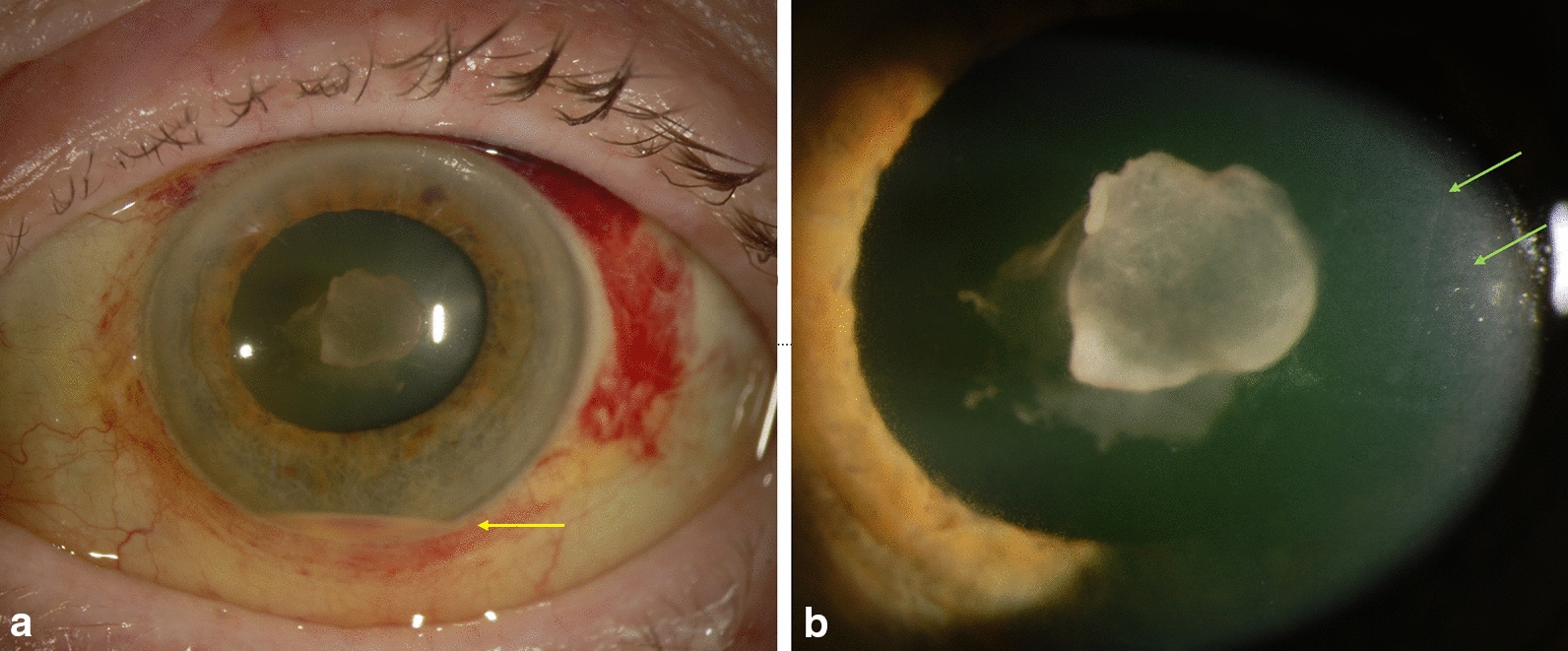
A. Slit lamp photograph of patient with endophthalmitis from *Granulicatella adiacens* shows layered ~ 1 mm hypopyon (yellow arrow) and fibrin plug on lens capsule. B. A higher magnification photograph shows the dense adhesion of the fibrin plug to the lens capsule. Note the corneal endothelial folds (Descemet’s folds, green arrows) indicating severe intraocular inflammation

An 84-year-old man presented with decreased vision, chemosis, hypopyon and vitreous inflammation, but no eye pain on March 7, 2013, three days after receiving an intravitreal bevacizumab injection (Fig. 1a). Pars plana vitrectomy with intravitreal injection of antibiotics was performed emergently. The vitreous inflammation gradually resolved over the next month.

#### Case 4

A 62-year-old woman received intravitreal bevacizumab injection in the affected eye on March 4, 2013 and in the fellow eye on March 6, 2013. On March 8, 2013, four days after her intravitreal injection, she presented with decreased vision, severe eye pain and vitreous inflammation, which was consistent with endophthalmitis. Emergent pars plana vitrectomy with intravitreal injection of antibiotics was performed. Postoperatively, she developed a dense vitreous hemorrhage, and the vision was hand motions at three feet immediately after surgery. The patient had been on the anticoagulant apixaban (Eliquis), which was subsequently discontinued by her cardiologist. On March 19, 2013, her vision declined to light perception, and she underwent a repeat vitrectomy and intravitreal vancomycin injection. A gram stain of the vitreous sample from the second surgery revealed gram-positive cocci, although cultures showed no growth. Her vision remained in the hand motions range due to persistent dense vitreous hemorrhage. On March 24, 2013, she was noted on B-scan ultrasound to have an inferotemporal retinal detachment, which was repaired with vitrectomy and silicone oil. Removal of silicone oil was performed on April 23, 2013. Her visual acuity improved to 20/200 and she was treated with three monthly aflibercept injections in April, May, and June 2013. She underwent cataract surgery on August 6, 2013. and her visual acuity eventually recovered to 20/25 in the affected eye by December 13, 2013.

### Prophylactic intravitreal vancomycin consideration and treatment

Because of the number of patients who had presented in a short period of time with severe, sight-threatening endophthalmitis, it was unclear how many more cases of endophthalmitis would develop as the cluster of cases was unfolding. For this reason, all patients who had received intravitreal bevacizumab injection that week were contacted by telephone (or via certified, overnight mail if unreachable by phone) on day four (March 8, 2013) and asked to return to the clinic for prompt re-examination. Options discussed included observation with close follow-up versus prophylactic intravitreal injection of antibiotic with coverage for gram-positive organisms. Between March 8 and March 10, 2013, 34 eyes were treated with prophylactic intravitreal vancomycin (1.0 mg/0.1 cc) after informed consent was obtained. One patient was allergic to vancomycin and was treated with oral moxifloxacin. One patient declined prophylactic antibiotic injection and elected close follow-up. The remaining five patients were not treated because they were unable to return to the clinic before day seven (March 11, 2013) and were noted to be unaffected by their follow-up exam.

### Public health notification and reporting

The Centers for Disease Control and Prevention (CDC), Georgia Department of Health, and the pharmacy were notified on March 7, 2013 (day three) and FDA MedWatch was notified on March 8, 2013 (day four), as it became apparent that a cluster of endophthalmitis cases was evolving. The American Society of Retina Specialists (ASRS) Adverse Event Reporting Section was also notified the following week. Patients previously scheduled to receive anti-VEGF injections in the afternoon clinic on March 6, 2013 and over the next two weeks were diverted to other retina centers while further investigation was undertaken. The pharmacy issued a nationwide voluntary recall of all bevacizumab unit dose syringes on March 15, 2013. Following an investigation into the outbreak, the CDC concluded that contamination was likely introduced during repackaging by the compounding pharmacy, where deficiencies in the pharmacy’s sterile compounding processes were documented.

### Microbiology identification of organism and sensitivities

Culture results from the vitreous aspirates of all four affected cases were positive for *Granulicatella adiacens.* The time to identification of the organism was seven days. Susceptibility testing later became available and revealed that the organism was sensitive to vancomycin, ceftriaxone and levofloxacin.

### Cluster definition and statistical analysis with binominal tail probabilities

Assessment of whether or not an outbreak is occurring and determination of the appropriate intervention requires a rigorous definition of a disease *cluster.* A *disease cluster* occurs when the number of endophthalmitis cases in a given batch of bevacizumab not only significantly exceeds what is to be expected under endemic rates of endophthalmitis for a single batch, but also significantly exceeds what is expected among all *N* batches of bevacizumab delivered in the United States within a given year. Under endemic rates, the probability *p*_*n*_ of *n* or more cases of endophthalmitis in a single batch of *M* doses of bevacizumab may be computed using binomial tail probabilities. The probability that at least one of those batches will result in *n* or more infections is ${\pi }_{n}=1-{\left(1-{p}_{n}\right)}^{N}$. The occurrence of *n* or more cases in a given batch is defined to be an endophthalmitis *cluster* if ${\pi }_{n}$ is small. The expected number of clusters of size *n* is $N\times {\pi }_{n}$.

To be conservative, binomial tail probabilities of *n* or more cases were computed based on a batch size of 70, the typical of the number of doses generated by the pharmacy from a single 100 mg vial of the drug. Table 2 gives binomial tail probabilities of *n* or more infections out of a batch of 70 doses for endophthalmitis incidence rates ranging from 0.02 to 0.05% (or 1 in every 2,000 to 5,000 injections). These estimates confirm that clusters from a single batch are rare: ~ 1 in 10,000 for 2 cases and 1 in 2 million for 3 cases (Table 2).

**Table 2:** Binomial tail probabilities of $\ge n$ number of cases of endophthalmitis in a batch of 70 bevacizumab injections

Number of infections (*n*)	Incidence	
	0.02% (1 in 5000)	0.05% (1 in 2000)
$\ge 2$	$9.57\times {10}^{-5}$	$5.90\times {10}^{-4}$
$\ge 3$	$4.34\times {10}^{-7}$	$6.62\times {10}^{-6}$
$\ge 4$	$1.45\times {10}^{-9}$	$5.58\times {10}^{-8}$

To estimate the likelihood of an endophthalmitis cluster in the context of intravitreal bevacizumab administered in clinical practice in the United States, we derived the number of intravitreal bevacizumab injections and batches as follows. The number of intravitreal bevacizumab injections given in the U.S. was estimated to be approximately 1,500,000, based on Medicare Provider Utilization and Payment Data for 2013 (1,080,635 fee-for-service Medicare beneficiaries [11], plus an additional 30% estimated Medicare-Advantage beneficiaries [12] and a conservative estimate of 10% of patients under the age of 65 in private plans). This corresponds to $N=\mathrm{21,428}$ batches of 70 bevacizumab injections per batch. With an estimated $N=\mathrm{21,428}$ batches of bevacizumab injections per year in the United States, the probabilities ${\pi }_{n}$ that at least one batch in the U.S. will result in *n* or more infections were computed under scenarios corresponding to the range of estimated incidences (Table 3).

**Table 3:** Probabilities ${\pi }_{n}$ of clusters exceeding various sizes in at least one batch among all those administered in the United States as a function of estimated incidence assuming that the number of batches $M=\mathrm{21,428}$

Number of infections (*n*)	Incidence	
	0.02% (1 in 5000)	0.05% (1 in 2000)
$\ge 2$	0.871362606	0.99999678
$\ge 3$	0.009256645	0.132251914
$\ge 4$	0.000031070	0.001194968

The probability of a cluster equaling or exceeding the four observed cases reported here is remarkably low, ranging from 0.003% to 0.1%, assuming the incidence of endophthalmitis to be 0.02% or 0.05%, respectively, indicating the significance of our cluster (Table 3). However, the probability of a cluster of two or more endophthalmitis infections in at least one batch in the U.S. annually is not as unlikely: there is an 87% chance of a cluster of two or more cases in the U.S. yearly assuming an incidence of 0.02%. With increasing numbers of bevacizumab injections each year, the above expression implies that the probabilities reported in Table 3 and the number of clusters of size *n* or more are expected to increase over time.

## Conclusion

In this report, we describe our clinical experience and detailed management of a concerning and rapid outbreak of four cases of endophthalmitis following intravitreal injections from a single lot of bevacizumab. Our intervention to quell the outbreak of endophthalmitis included offering prophylactic intravitreal vancomycin, given the severe vision impairment and initial concerns of the potential for permanent vision loss without prompt intervention, and risk mitigation measures. Collaboration with the CDC, ASRS, and Georgia Department of Health were essential to the management of this infectious disease cluster, which included evaluation of eye clinic protocols for intravitreal injection, infection control precautions and assessment of environmental precautions, as well as assessment of the compounding pharmacy. Our findings raise several considerations related to protocol on managing these scenarios, for which there currently is no definitive consensus.

Emergent vitreous tap for Gram stain and culture, in conjunction with intravitreal vancomycin and ceftazidime injections or pars plana vitrectomy with intravitreal antibiotic injection remains the mainstay for the immediate treatment of post injection endophthalmitis. Prophylactic antibiotic injection may be considered to limit the outbreak. One of the primary challenges in managing an endophthalmitis cluster is the real-time identification of an outbreak. For instance, does the identification of a second case constitute a cluster or should providers wait until a third case occurs to initiate an outbreak-specific management strategy? Our statistical analysis suggests that while clusters from a single batch are rare, the likelihood of clusters occurring in a particular year given the scale of all injections in the USA is perhaps greater than one might expect (87% chance of a cluster ≧2 cases and a 1% chance of a cluster ≧3 cases yearly, Table 3). Had effective prophylactic injections been administered immediately following the second case in this series, two cases of endophthalmitis could have potentially been prevented. However, estimation of the number of additional cases of endophthalmitis once an outbreak is identified has limitations because batch contamination may not be uniform and patient susceptibility can be variable. Moreover, there are risks associated with additional antibiotic injection as well.

A conservative recommendation would potentially include immediate prophylactic antibiotic injection following the second endophthalmitis case from a given batch. This approach may result in over-treatment; however, the risks of potential vision loss from endophthalmitis must be weighed against the risks and costs of prophylactic antibiotic injection. For example, there have been recent reports of hemorrhagic occlusive retinal vasculitis (HORV) associated with intraocular vancomycin injection leading to severe vision loss [13, 14]*.* HORV was not observed in the patients receiving intravitreal vancomycin injection in our series. Ultimately, the choice of appropriate prophylactic antibiotic should be based on available culture results weighted against the risks of adverse events related to the antibiotic itself.

The unpredictable nature of an outbreak presents unique challenges as it requires a rapid response and can elicit anxiety among patients and the health care staff, and potentially lead to economic and social disruption. The World Health Organization has identified five essential best practices when faced with an outbreak: building public confidence, early announcement, transparency, respect for public concerns, and planning in advance [15]. Presently there are no guidelines specific to an endophthalmitis outbreak for ophthalmology providers to follow. Given the growing number of intravitreal injections in the United States, our ability to quickly and effectively manage an emerging outbreak situation is paramount.

**Fig. 2:**
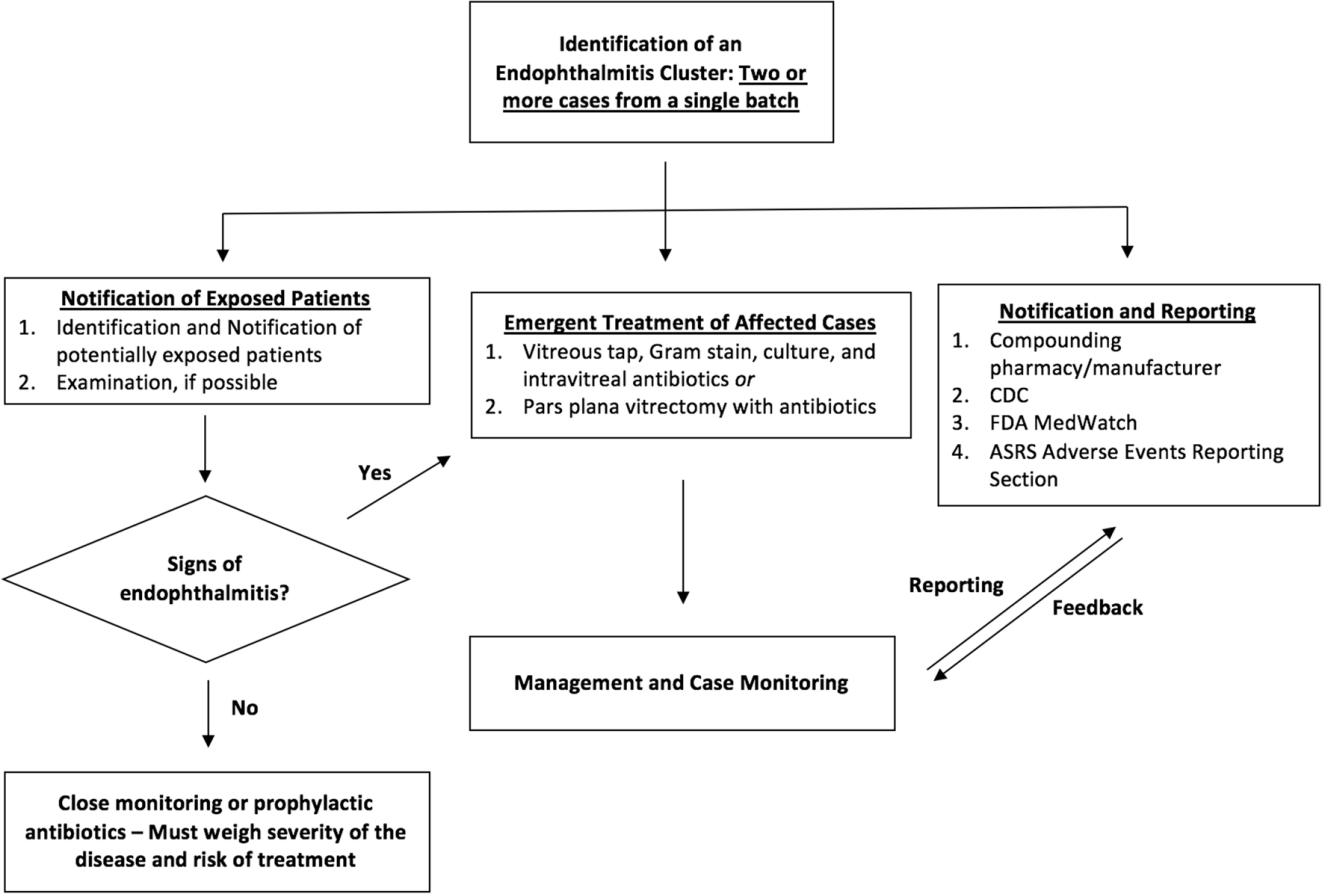
Proposed process diagram for management of an endophthalmitis outbreak when a cluster is suspected

Based on our experience in this outbreak and others reported in the literature, future recommendations for ophthalmologists faced with these dilemmas could include the following: 1) Treatment of affected cases with urgent vitreous tap for Gram stain and culture or pars plana vitrectomy and intravitreal injection of antibiotics; 2) Prompt explanation to the patients, notification to the pharmacy and such appropriate agencies including FDA MedWatch, CDC, Department of Health, and the ASRS Adverse Events Reporting Section; 3) Close follow-up of the remaining patients versus prophylactic injection of appropriate antibiotic based on Gram stain and culture results, while weighing the risks of antibiotic injection and the ability of patients to return for close follow-up (Fig. 2).

## Data Availability

Patient data are presented in the body of this manuscript and specific data can be made available via the corresponding authors.

## Prior presentation

This abstract was presented at the Association for Research in Vision and Ophthalmology Annual Meeting in Vancouver, Canada, April 29, 2019.
